# Editorial: Discovery of novel plant-derived compounds with antibacterial actions against antibiotic-resistant bacteria, volume II

**DOI:** 10.3389/fmicb.2022.1027679

**Published:** 2022-09-08

**Authors:** Ren-You Gan, Hua-Bin Li, Harold Corke, Hongshun Yang

**Affiliations:** ^1^Singapore Institute of Food and Biotechnology Innovation, Agency for Science, Technology and Research, Singapore, Singapore; ^2^Guangdong Provincial Key Laboratory of Food, Nutrition and Health, Department of Nutrition, School of Public Health, Sun Yat-sen University, Guangzhou, China; ^3^Biotechnology and Food Engineering Program, Guangdong Technion-Israel Institute of Technology, Shantou, China; ^4^Faculty of Biotechnology and Food Engineering, Technion-Israel Institute of Technology, Haifa, Israel; ^5^Department of Food Science and Technology, National University of Singapore, Singapore, Singapore; ^6^National University of Singapore (Suzhou) Research Institute, Suzhou, China

**Keywords:** natural products, antibacterial effect, virulence factors, molecular mechanism, nanoparticles, antibiotic resistance

The wide and even abuse of antimicrobials in medicine and agriculture has led to severe widespread antimicrobial resistance, causing reduced effectiveness or even ineffectiveness of many antibacterial agents (Hobson et al., 2021). Therefore, it is essential and urgent to discover novel antibacterial agents that can effectively fight against antibiotic-resistant bacteria. Nature is a reservoir for diverse antimicrobial agents. Our and other recent research indicates that numerous medicinal plants, spices, and their bioactive compounds possess antibacterial properties (Saleem et al., 2010; Moloney, 2016; Zhang et al., 2019, 2021; Kim et al., 2020; Porras et al., 2021; Song et al., 2021). In addition, some phytochemicals have been reported to inhibit microbial virulence factors, such as bacterial biofilm, quorum sensing, and bacterial toxins, with the effective concentrations lower than their minimum inhibitory concentrations (MICs) (Ferro et al., 2016; Silva et al., 2016; Zhang et al., 2018, 2020; Wu et al., 2019; Farha et al., 2020; Liu et al., 2020; Melander et al., 2020; Tahrioui et al., 2020; Zamuz et al., 2021). Thus, plant-derived antibacterial compounds represent a category of promising alternatives to traditional antibiotics, while their efficacy and underlying molecular mechanism against antibiotic-resistant bacteria have not yet been entirely understood.

This current Research Topic is aimed to underpin the recent research progress on the antibacterial and antivirulence properties and related molecular mechanisms of novel plant-derived compounds against antibiotic-resistant bacteria, their micro/nano-encapsulation to enhance the antibacterial activities, as well as their potential applications as food preservatives or antibiotic alternatives in clinic and animal feed. In the collection, we published four excellent papers.

Wen et al. reported a novel antibacterial compound moringin [4-(α-L-rhamnosyloxy) benzyl isothiocyanate] against *Listeria monocytogenes*. It is the bioactive form of glucomoringin, an isothiocyanate found in the *Moringa oleifera* seeds. Moringin was found to exhibit strong antimicrobial activity against *L. monocytogenes*, with a MIC of 400 μM. Further transcriptome data revealed that moringin treatment regulated the expression of genes related to the biosynthesis of cell wall and membrane, phosphotransferase system, DNA binding, energy metabolism, and oxidative stress. The authors, therefore, proposed that moringin could induce the death of *L. monocytogenes* by damaging the cell wall and cell membrane integrity, stimulating oxidative stress, and interfering with energy metabolism and DNA replication. This study indicates that isothiocyanates can be a novel type of antibacterial compounds, and the antibacterial effects and related molecular mechanisms of other isothiocyanate compounds should be interesting for exploration.

Chlorogenic acid (CGA) is a multifunctional phenolic acid existing in many functional plants, especially rich in *Eucommia ulmoides, Lonicera japonica*, and the genus *Ilex*. The outer membrane (OM) has been reported as the main target for the antibacterial effect of CGA, while how CGA damages the bacterial cell wall remains not very clear. In this study, Yang et al. clarified the antibacterial mechanism of CGA against *Salmonella*. It was demonstrated that sub-lethal doses of CGA (MIC = 6.25 mg/L) could evidently enhance the OM permeability, leading to the release of soluble proteins, and the bacterial cells exhibited increasing deformation, shrinkage and fluidity, supporting the impairment of cellular membrane integrity, which should be one non-specific mechanism for many natural products against bacteria, in agreement with our recent result on 1'-acetoxychavicol acetate (Zhang et al., 2021), and this may be exempted from antimicrobial resistance due to non-specific molecular targets.

Another study by Yoshino et al. discovered that *Foeniculum vulgare* (fennel), a spice as well as a medicinal plant, exhibited dual inhibitory activity against *Porphyromonas gingivalis*. The *n*-hexane-extracted fennel (HEF) exhibited a rapid bactericidal effect toward *P. gingivalis*. It was found that HEF at a low dose (8 μg/mL) led to the formation of protruding nanostructures composed of out membrane vesicle (OMV)-like particles, while it could induce bacteriolysis with overproduction of OMVs with unusual surface properties at a high dose (64 μg/mL). Further mechanism studies indicated that HEF treatment could deprive two outer membrane transporter proteins, RagA and RagB, which are necessary for nutrient intake for the bacteria. On the other hand, the authors also found that HEF had gingipain-inhibitory activity toward both arginine-specific (Rgps) and lysine-specific (Kgp) gingipains in cell models. Finally, it was demonstrated that the petroselinic acid, an isomer of oleinic acid found in HEF, was a major bactericide as well as a gingipain inhibitor. This study indicates that certain long-chain fatty acids can be potential novel antibacterial compounds, which might be generally ignored considering the conventional natural product extraction methods based on methanol or ethanol solutions.

Essential oils are promising antibiotic alternatives. Ambrosio et al. investigated the effect of citrus essential oil (EO) and its microencapsulated formulations (MFs) on *in vitro* fermentation kinetics of pig gut microbiota. It was found that the EO and MFs affected the abundance of ileal and colonic microbiota, with reduced phylogenetic diversity and altered composition. In addition, MFs exhibited more pronounced effects than EO, suggesting microencapsulation could enhance the antibacterial effect of EO. Moreover, MFs could stimulate the production of lactate and short-chain fatty acids during *in vitro* fermentation, and the authors suggested that the wall material of microcapsules like chitosan and modified starch might provide an additional carbon source with prebiotic functioning, stimulating the growth and metabolic activity of colonic bacteria. Therefore, it may exist a synergistic effect of natural products and their capsule wall materials on gut microbiota, which needs further investigation in the future.

In conclusion, plant-derived compounds are promising candidates for novel antimicrobial agents. According to the literature, the A^*^STAR Natural Product Library (NPL), currently under the Singapore Institute of Food and Biotechnology Innovation (SIFBI), A^*^STAR, has about 160 K natural products, including 37,000 plant specimens and 120,000 environmental microbial strains (Ng et al., 2018). The A^*^STAR NPL is a valuable resource for the discovery of novel antibacterial agents with potential applications. In the future, more NPLs all over the world can be established to collaborate with each other to explore more novel natural compounds with antibacterial as well as other bioactivities.

## Author contributions

R-YG wrote the manuscript draft. H-BL, HC, and HY edited the manuscript. All authors approved the final version of the manuscript for publication.

## Conflict of interest

The authors declare that the research was conducted in the absence of any commercial or financial relationships that could be construed as a potential conflict of interest.

## Publisher's note

All claims expressed in this article are solely those of the authors and do not necessarily represent those of their affiliated organizations, or those of the publisher, the editors and the reviewers. Any product that may be evaluated in this article, or claim that may be made by its manufacturer, is not guaranteed or endorsed by the publisher.
